# Interpersonal Variations in Gut Microbiota Profiles Supersedes the Effects of Differing Fecal Storage Conditions

**DOI:** 10.1038/s41598-018-35843-0

**Published:** 2018-11-26

**Authors:** Caspar Bundgaard-Nielsen, Søren Hagstrøm, Suzette Sørensen

**Affiliations:** 1 Centre for Clinical Research, North Denmark Regional Hospital, Hjørring, Denmark; 2 Department of Clinical Medicine, Aalborg University, Aalborg, Denmark; 3 Department of Pediatrics, Aalborg University Hospital, Aalborg, Denmark

## Abstract

Due to ease of acquisition, fecal samples are often used in studies investigating gut microbiota. Improper handling of these samples can lead to bacterial growth and alter bacterial composition. While freezing samples at −80 °C is considered gold standard, this is not suitable for studies utilizing self-sampling by lay participants or field studies. Thus to effectively prevent bacterial growth, techniques that allow efficient fecal storage outside laboratory facilities are needed. Fecal samples were collected from three donors. From each donor feces, 45 samples were collected and stored either freshly frozen at −80 or −20 °C, or in three separate storage buffers at room temperature or 4 °C for 24 or 72 hours. Bacterial composition was analyzed using Illumina amplicon sequencing of the V4 region of the 16 S rRNA gene. While storage conditions did affect bacterial composition and diversity compared to storage at −80 °C, the variation between donors superseded the variations introduced by storage. Samples stored at −20 °C most closely resembled those stored at −80 °C. When investigating variations in bacterial composition between separate study populations, fecal samples can efficiently be stored in −20 °C freezers or in one of the presented storage buffers, without severe alterations in bacterial composition.

## Introduction

Careful handling of biological samples is essential to avoid introduction of bias into the results. This is especially true for bacteria that may continue to grow during storage, changing the bacterial composition of samples^1–3^. Maintenance of bacterial composition is essential for studies investigating the role of gut bacteria in disease development. These gut bacteria, along with viruses, archaea and fungi, are collectively known as the gut microbiota, and has been implicated in maintenance of health^4^. Dysbiosis of the gut microbiota has conversely been associated with several disorders like infectious and autoimmune diseases^5–7^, obesity^8,9^, affective and neurodevelopmental disorders^10–12^.

Bacterial composition in feces is regularly used as a representative for investigation of gut microbiota, and is mapped through sequencing of the 16 S ribosomal RNA (rRNA) gene^13,14^. Several bacterial species, including *Faecalibacterium*, normally found in the human gastrointestinal system, are anaerobic bacteria whose growth is severely limited when exposed to air. In contrast, the growth of aerobic bacteria may be enhanced following defecation, and the subsequent altered bacterial composition may introduce severe bias to gut microbiota studies^1^. To prevent bacterial growth following collection, the gold standard consists of immediate freezing of feces at −80 °C or in liquid nitrogen^2,15,16^. This procedure may not always be readily available since many studies rely on self-sampling by the involved study participants, where fecal samples are stored in domestic freezers^2,17^. These samples may be exposed to temperature fluctuations due to automatic defrost cycles, frost accumulations and partial thawing during transport to the research fascilities^17^. Still other studies rely on sample collection in areas without access to proper freezing facilities^14^. One way to overcome these challenges is to use storage buffers or reagents that may aid in stabilizing the fecal samples at room temperature. However, for these to function as proper replacements for freezing, it is crucial that the bacterial composition is not compromised or altered during the handling period. Good results have been obtained using a number of storage conditions, including −20 °C^18–22^, OMNIgene^®^•GUT^16,17,23^, Fecal Occult Blood Test cards^15,24^, FTA cards^®^^17,21^ or >95% but not < 95% ethanol^17,21,22^. Several studies have similarly investigated the commonly available RNAlater^®^ buffer, but with mixed to negative results on the ability to sustain a microbiota profile^15–19,21,22,25^. Not all of the storage methods are, however, easily usable for lay participants which may introduce sampling variations, while other like ethanol can introduce transport restrictions^21^. PSP buffer is marked as a collection buffer that can be directly applied to the subsequent DNA extraction protocol. A study by Wu *et al*.^26^ showed that Stool DNA Stabilizer from the PSP^®^ Spin Stool DNA Plus Kit (PSP buffer) was able to sustain gut microbiota profile effectively for up to 48 hours. Another study investigated the effects of DNA/RNA shield^TM^, and found that the buffer was effective at maintaining microbiota profiles and DNA quality in sheep^27^. While positive results have been obtained with DNA/RNA shield and PSP buffer, they have not adequately been compared to other methodologies usable by lay participants, like domestic freezers. Here we aimed to investigate differing storage methodologies suitable for self-sampling by lay participants, and their effects on bacterial compositions in fecal samples.

## Results

**Figure 1:**
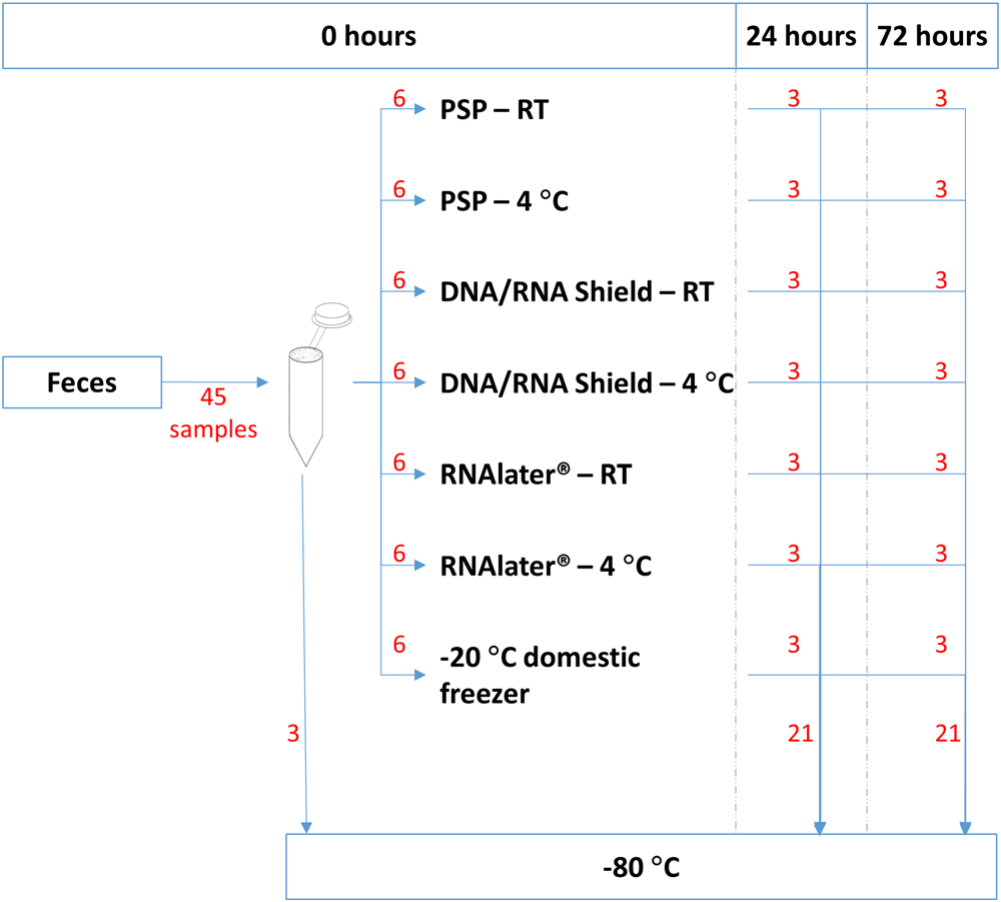
Overview of storage conditions for each of the three separate donor feces. 45 fecal samples were obtained from each donor feces. The samples were processed in triplicates and either stored directly at −80 °C, at −20 °C for 24 or 72 hours, or in one of the following buffers: DNA/RNA Shield, PSP Buffer, or RNAlater at 4 °C/RT, prior to freezing at −80 °C. Red numbers indicate number of samples that progress to this storage methodology.

This study investigated how storage of fecal samples in DNA/RNA shield, RNAlater, PSP buffer or frozen at −20 °C affected the resulting DNA output and bacterial composition compared to −80 °C. A total of 135 fecal samples were investigated, originating from three different donors, as illustrated in Fig. 1. DNA output was evaluated using yield, purity, and integrity of the extracted DNA. Effects on bacterial composition and diversity were investigated by 16 S rRNA gene sequencing. Following quality filtering and chimera removal, 2,906,405 16 S rRNA sequence reads were obtained, corresponding to a mean number of 21,529 ± 4,594 reads per sample. The mean number of reads per donor were 18,763 ± 2,878, 20,857 ± 3,721, and 24,967 ± 4667 for donor A, B, and C respectively. A total of 1,060 unique Operational Taxonomic Units (OTUs) were identified, with 95.5% being identified on the phylum taxonomic level, 80.1% on family level, 54.0% on genus level and 0.1% on species level. Importantly, all samples produced higher reads and OTU counts compared to negative controls, and comparable or higher number of reads and OTU compared to the anaerobic based positive control sample. A rarefaction curve was produced showing a good sequencing coverage (See Supplementary Images Section 1.1).

### DNA levels increase in two of three storage buffers compared to freezing, probably due to bacterial growth

**Figure 2:**
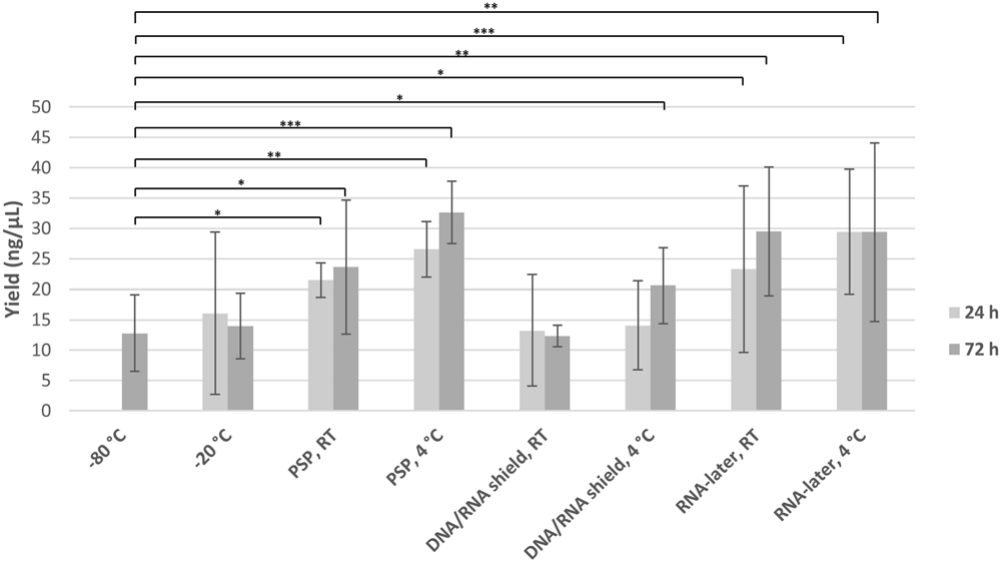
DNA yield. Yield of DNA from each storage condition of fecal samples, as measured using Qubit^TM^ Fluorometric Quantification.

To investigate the effects of fecal storage methodologies on bacterial growth, we measured the quantity of DNA following extraction. DNA yields were comparable for samples stored at −80 °C, −20 °C, and in DNA/RNA Shield. However, a higher DNA yield was observed for samples stored in PSP buffer or RNAlater (p < 0.05, Fig. 2), indicating that these buffers may lead to increased bacterial growth. Neither temperature nor storage time affected DNA yield significantly.

### DNA integrity and purity is not affected by use of storage buffers

**Figure 3:**
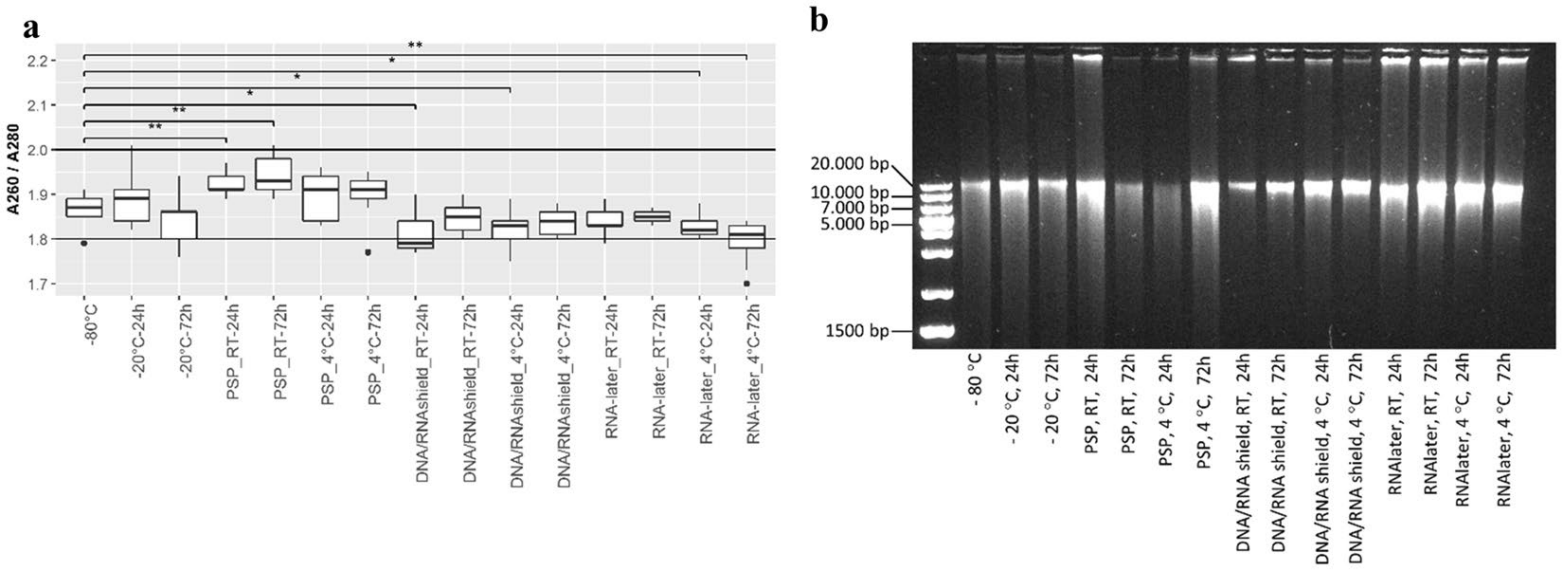
Integrity and purity of DNA extracted from each storage condition. (**a**) Purity of DNA extracted from fecal samples from each storage condition as measured by spectrophotometry. *p < 0.05. (**b**) Representative 1% agarose gel showing the effect of the different storage conditions on the integrity of DNA extracted from stool from donor A. Comparable results were observed for all replicates and donors from donor A, B and C. All agarose gels can be observed in Supplementary Images Section 1.3. RT: Room temperature.

For proper downstream analyses of bacterial DNA, it is important that DNA has good purity and is relatively intact. The purity of extracted DNA was measured using the 260/280 nm absorbance ratio (Fig. 3a). While variations did occur, all storage conditions maintained DNA with an A_260/280_ OD ratio localized between 1.8 and 2.0. To ensure that DNA was not degraded during storage of DNA extraction, DNA integrity was investigated using agarose gel electrophoresis. For all samples, DNA was visible as a smear with the strongest signal observed between 10,000 and 20,000 bp (Fig. 3b). No visible differences in DNA fragment size were observed between storage conditions or durations.

### Interpersonal differences in bacterial composition are larger than differences introduced through differing fecal storage conditions

**Figure 4:**
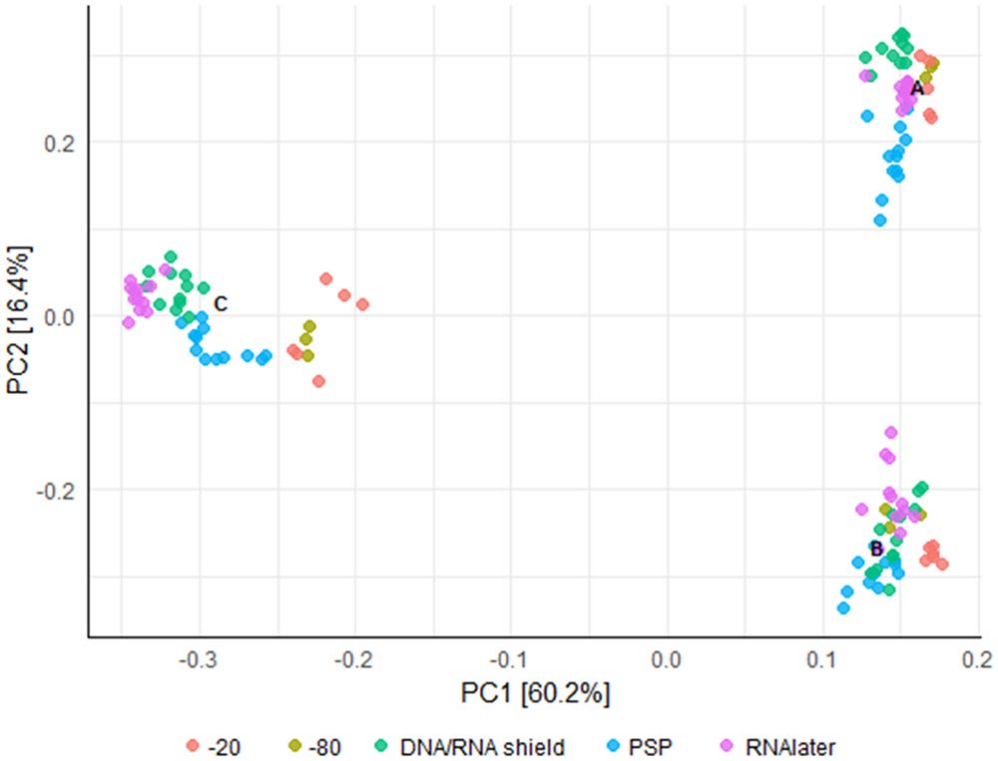
Differences in bacterial composition between storage conditions and the individual donors A, B and C. Clustering based on donor identity was seen using PCA plot using Hellinger Distance. Minor storage specific clustering was observed, but this was superseded by interdonor variation. PC1 explains 60.1% of variability between samples whereas PC2 explains 16.5%. All samples were frozen at −80 °C for a minimum of 24 hours following end of storage. PC: Principal component.

**Figure 5:**
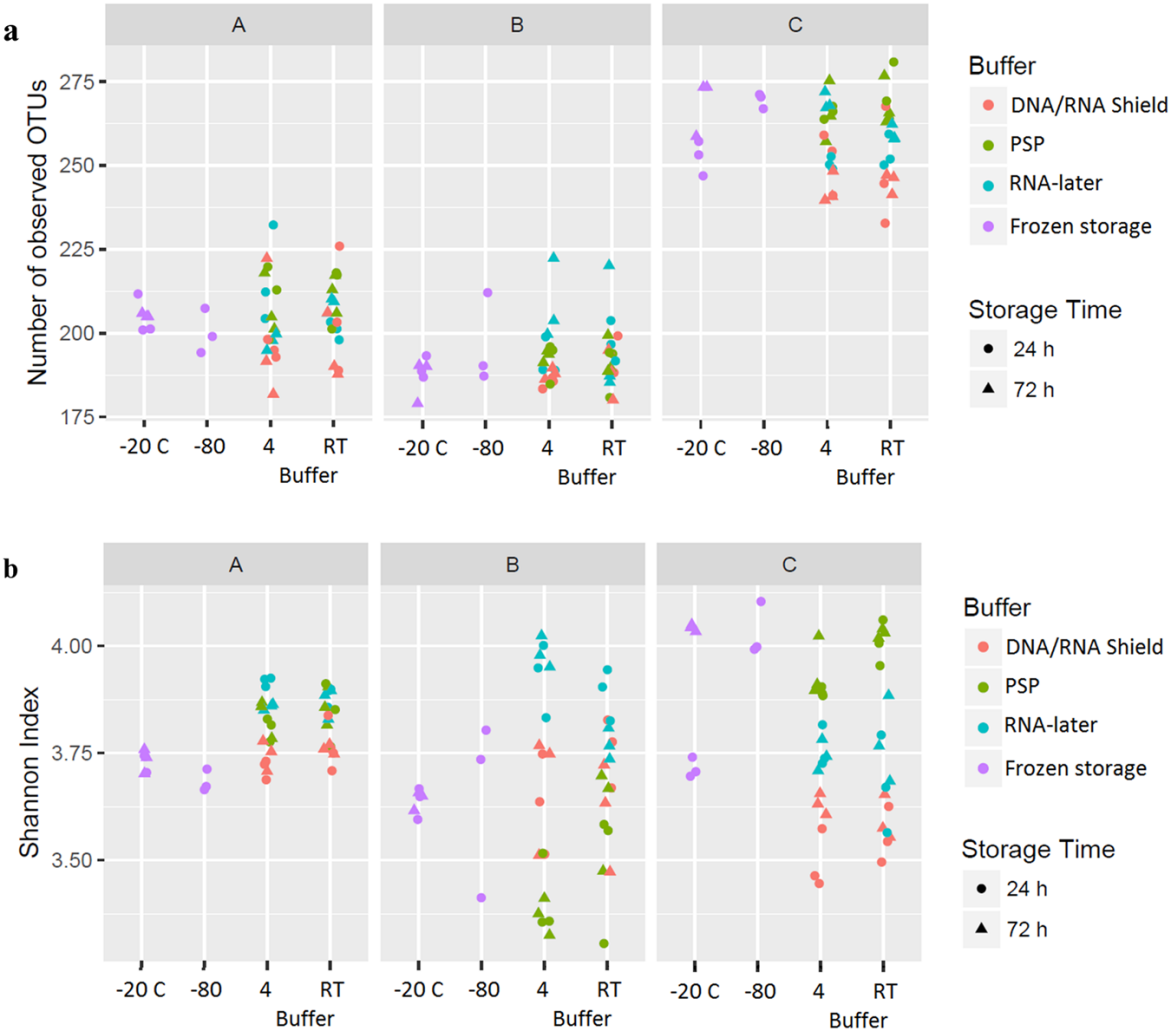
Bacterial α-diversity in feces from different study participants stored under differing conditions. The bacterial diversity was evaluated based on (**a**) number of different OTUs observed in each sample, and (**b**) the Shannon Diversity Index.

**Figure 6:**
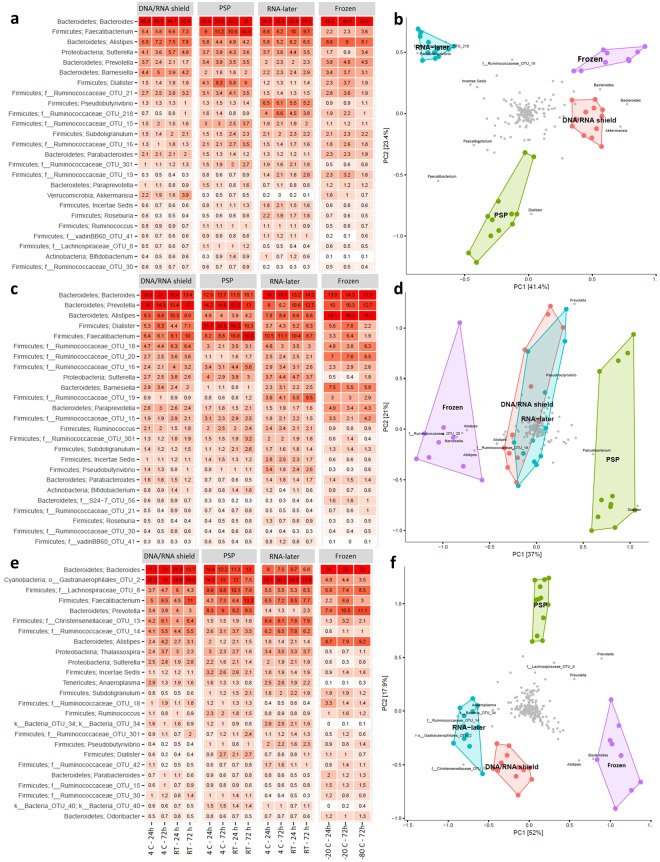
Bacterial composition of differing study participants and storage methodologies. Differences in bacterial compositions between individual storage conditions in study participant A (**a**,**b**), B (**c**,**d**) and C (**e**,**f**). Image (**a**,**c**,**e**) consists of heat maps representing the 25 most common genera/OTUs in different fecal storage conditions in the individual donor. Numbers describe the percentage of total reads that consists of this specific OTU. Each name consists of phylum name followed by a more specific genus name. If no genus name was available, the best assignment is shown. Image (**b**,**d**,**f**) visualize PCA plots. Within individual donors, storage methodology dependent clustering was observed using Hellinger Distance. Samples stored at −20 °C and −80 °C clustered together and were therefore combined. PC1 covers 41.1%, 37% and 52% of variation while PC2 represent 23.4%, 21% and 17.9% of variation respectively. Frozen: samples stored at either −80 °C or −20 °C, PC: Principal component; RT: Room temperature.

Storage of fecal samples outside −80 °C may favor growth of individual bacterial genera following delivery that may skew bacterial composition. We therefore evaluated the effects of storage conditions on bacterial composition and diversity, using a Principal Component Analysis (PCA) of Hellinger Distance between OTU abundances (Fig. 4)^28^. We found that β diversity clearly superseded variations introduced by differing storage conditions in all donors. We next investigated factors influencing α diversity of samples, using bacterial richness, diversity and variability of all samples within donors. No effects of storage were found in bacterial richness (p < 0.05, Fig. 5a), whereas a non-significant (p < 0.05) altered Shannon diversity index was observed in samples stored in buffers compared to samples stored at −80 °C, most noticeable in samples from donor B and C (Fig. 5b). Within individual donors, samples stored at −20 °C and −80 °C clustered together and were combined. Samples stored using RNAlater, DNA/RNA shield, or PSP buffer, clustered based on buffer type, separate from frozen samples. (Fig. 6b,d,f). To investigate which bacteria were affected by differing storage conditions, the 25 most abundant genera for each donor were identified (Fig. 6a,c,e). For all donors *Faecalibacterium* was more abundant while *Alistipes* was less abundant in storage buffers compared to samples stored at −80 °C and −20 °C. This was especially evident in samples stored in PSP buffer in donor A and B. Interestingly, for donor C, an OTU from the phylum *Cyanobacteria* was observed to constitute a large percentage of the total OTU abundance in all samples stored in storage buffers but not for samples frozen at −20 °C or −80 °C. Importantly, for all samples, the variations introduced by storage methodology were larger than differences between individual triplicates (see Supplementary Images Section 1.2).

## Discussion

Proper handling of fecal samples prior to DNA extraction is imperative for studies on the effects of gut microbiota on health, as biases can easily be introduced depending on choice of sampling and storage methodology^2^. In this study, we investigated the effects of different storage conditions on composition of bacterial communities in fecal samples from three healthy donors using Illumina sequencing of the V4 region of the 16 S rRNA gene.

For all samples, the variations in bacterial composition between individual donors, clearly superseded variations introduced by storage condition or duration. This is in line with other studies which reported that the bacterial signature of donor identity was maintained despite differing storage methodologies^2,20,22^. This substantial β diversity is most likely caused by differences in donor specific factors including diet, lifestyle^29,30^ and genetics^31,32^, which may hide more subtle variations in bacterial composition. To better determine the effects of differing storage methodologies, bacterial composition and diversity was assessed separately for each donor. We found, that storage conditions, but not durations affected α diversity of samples. The bacterial composition of fecal samples stored at −20 °C was highly correlated with that of feces stored at −80 °C, confirming previous reports that storage at −20 °C is effective for studies investigating microbiota of feces^17,21,22^. All storage buffers had minor variations in α diversity compared to samples stored at −80 °C, but no clear superior or inferior buffer could be recognized. To determine the cause of differing α diversities and DNA yields in buffers compared to frozen samples, we looked at composition of individual bacterial genera. Especially the bacterial genus *Faecalibacterium* was found to be more abundant in fecal samples stored in buffers compared to feces stored at −80 °C. This bacterial genus has previously been shown to be insufficiently stabilized by other buffers^16^, and thus we suggest that studies investigating these bacteria should be conducted with care. While the use of storage buffers did result in minor variations in α diversity, all storage conditions were capable of maintaining the interindividual variations.

During DNA extraction, we found that it was difficult to fully separate RNAlater and feces using centrifugation, an observation that has also previously been reported^27,33^. Since RNAlater can influence subsequent cell lysis during DNA extraction^33^, this could affect the microbiota profile. In two out of three participants, we observed a slightly reduced composition of bacteria belonging to the *Bacteroidetes* phylum and especially the genus *Prevotella* in samples stored in RNAlater, similar to observations by Hale *et al*.^21^ and Sinha *et al*.^24^ but in contrast to results obtained from Vogtmann *et al*.^34^. While RNAlater was suitable for distinguishing participant identity after storage, it was not optimal for storage of feces for microbiota studies. PSP was found to increase the proportion of bacterial genera belonging to the phylum* Firmicutes*. A similar observation was observed by Wu *et al*.^26^, who suggested that PSP enables increased recovery of *Firmicutes*. The implications of this observation are not clear and need to be investigated in future studies. Finally, DNA/RNA shield had overall a bacterial composition close to that of frozen samples. This is not comparable to the results by Menke *et al*.^27^ who found a significantly lower diversity of bacteria in sheep fecal samples stored in DNA/RNA shield compared to frozen samples. This discrepancy may however, be due to different core bacterial microbiotas between human and sheep. Overall, while each buffer possess limitations, all variations caused by buffers were superseded by participant identity.

Bead beating is normally utilized in microbiota studies, to enable extraction of DNA from bacteria that are difficult to lyse, like Gram-positive bacteria^25,35^. Excessive bead beating may however, result in DNA shearing and a reduced recovery of vulnerable bacteria^35,36^. We therefore analyzed the integrity of the extracted DNA. The majority had a length located between 10,000 and 20,000 bp, which is in agreement with previous reports of bacterial DNA sizes from freshly extracted feces^25,36^. Overall, all storage conditions produced a high DNA output with good purity and integrity, suitable for Illumina sequencing of the 16 S rRNA gene.

This study has certain limitations. First, the microbiota composition throughout an entire stool sample has been shown to be heterogeneous, which may introduce bias^18^, and effective homogenization of feces prior to sample collection has been recommended^25^. However, this study aimed to investigate techniques feasible for lay participants, where homogenization is seldom possible^18^. Despite the lack of homogenization, our study did not find separate clustering of triplicates based on location but rather based on donor first, followed by storage methodology. Secondly, only three study participants were included, which limits the power of this study. The aim of this study was, however, to compare how different storage conditions affected the bacterial composition of fecal samples. For this purpose, testing the same storage conditions of several replicate samples from the same original feces (45 samples from each participant for a total of 135 fecal samples), was judged to more effectively assess the effects of storage compared to few samples from several participants. Using several samples from several participants would however, increase the power of this study. Finally, we did not compare the different storage conditions to a freshly extracted sample. This was precluded since conditions compatible with home sampling was the main scope of our study. Importantly, however, a previous study by Fouhy *et al*.^37^, found that storage of fecal samples at −80 °C maintained a microbiota composition similar to freshly extracted samples.

Despite these limitations, this study has a number of strengths. We employed a structured study design with comparison of a high number of different storage conditions. Importantly, the methods are of low cost and fully suitable for home collection. These results are therefore very relevant for large-scale microbiota studies. Additionally, in order to secure the validity of our results and avoid misinterpretation, we included triplicate experiments, which resulted in a large sample size. Finally, all sequencings have been compared to an anaerobic digester that covers several differing bacterial phyla and genera, thus ensuring a good sequencing coverage.

## Conclusions

While previous studies have investigated the effects of storage on the bacterial composition of fecal samples, the current study provides an in-depth investigation of the effects of storage methodologies suitable for home sampling by study participants compared to the gold standards of laboratory facilities.

Differing storage methodologies did introduce minor variations in bacterial composition, but the β diversity clearly superseded the variations introduced by storage. Thus, all investigated storage methodologies are suitable for stabilizing fecal samples for microbiota studies, with feces stored at −20 °C most closely resembling feces stored at −80 °C.

## Methods

### Sample collection and storage conditions

Fresh feces was collected from three healthy anonymous donors. As each storage condition was tested in triplicates, 45 samples (200 mg ± 50 mg of feces per sample) per donor was analyzed, resulting in a total of 135 fecal samples being analyzed for this study (Fig. 1). Briefly, three fecal samples were immediately frozen at −80 °C, while another three samples were frozen at −20 °C for 24 or 72 hours in a domestic freezer with manual defrost(EUC19001W, Electrolux). The remaining samples were stored in either DNA/RNA Shield^TM^ (Zymo Research, USA), Stool DNA Stabilizer from the PSP^®^ Spin Stool DNA Plus Kit (PSP buffer, Stratec Molecular, Germany), or RNAlater^®^ (Invitrogen, Thermo Fisher Scientific, USA) at room temperature or 4 °C, for either 24 or 72 hours. Subsequently, all samples were collectively stored at −80 °C to rule out bias due to differences in low temperature exposure.

### DNA extraction

Bacterial DNA was isolated from fecal samples using the QIAamp^®^ Fast DNA Stool Mini Kit (QIAGEN^®^, Germany) automated on a QIAcube^®^ (QIAGEN), according to manufacturer’s protocol with the addition of a manual pretreatment step to enhance lysis of gram positive bacteria. In this pretreatment step, fecal samples containing storage buffer were centrifuged for 5 min at 14,500 x g, the supernatant was discarded, and all samples, including frozen samples, were resuspended in InhibitEX^®^ lysis buffer. Bead beating was performed using a single 5 mm stainless steel ball (QIAGEN) on a TissueLyser LT (QIAGEN) for 4 min at 30 Hz. This was followed by lysis at 95 °C for 5 min, and 200 μL of the resulting lysate solution was transferred to spin columns continuing with the standard protocol.

Purity of the extracted DNA was evaluated spectrophotometrically, with a Nanodrop^TM^ Lite (Thermo Fisher Scientific) using the A_260/280_ OD ratio. The concentration of DNA was measured using the Qubit^TM^ HS Assay (Thermo Fisher Scientific). Finally, DNA integrity was evaluated by agarose gel electrophoresis.

### 16S rRNA gene sequencing

Bacterial 16S rRNA amplicon sequencing targeting the V4 variable region, was performed by DNAsense ApS (Denmark), and followed a modified version of an Illumina protocol^38^. An initial amplicon PCR and clean-up was performed as described by Albertsen *et al*.^35^, using the V4 primers (5′-GTGCCAGCMGCCGCGGTAA and 5′-GGACTACHVGGGTWTCTAAT^39^), and 35 cycles of amplification. This was followed by an index PCR and clean-up^38^. Finally, samples were pooled and sequenced on a MiSeq^TM^ (Illumina^®^, USA) as previously described^40^, but with the addition of 20% PhiX control library (Illumina, USA) to measure error rate during sequencing, a negative control (nuclease-free water) to eliminate background, and a positive control (complex sample obtained from an anaerobic digester system) to ensure efficient sequencing.

### Statistics and data analysis

Quality of sequencing reads were analyzed using FastQC (Babraham Bioinformatics, UK). Forward reads were quality trimmed using Trimmomatic v 0.32^41^ utilizing settings SLIDINGWINDOW:5:3 and MINLEN:250 to remove reads with a Phred Score below 20 and discard reads shorter than 250 bp. The reads were next dereplicated and formatted for use in the UPARSE workflow^42^. The first 250 bp of all reads were clustered using the usearch v. 7.0.1090 -cluster_otus command with default settings. OTUs were clustered based on 97% identity and chimeras removed using the usearch v. 7.0.1090 –usearch_global command with –id 0.97. Taxonomy was assigned using the RDP classifier^43^ as implemented in the parallel_assign_taxonomy_RDP.py script in QIIME^44^ using the MiDAS database v. 1.20^45^.

Data analysis was performed in R^46^ through the Rstudio IDE (http://www.rstudio.com/) using the ampvis2 package v.2.3.11^35^, as well as Microsoft Office Excel 2013. α diversity was determined using OTU richness and Shannon Diversity Index as implemented in the amp_alphadiv command of the ampvis2 packet in R. β diversity was determined using PCA clustering and heat maps in the ampvis2 package. PCA plot using Hellinger Distance was performed using the amp_ordinate function, while heatmaps displaying the composition of the 25 most common OTUs were generated using the amp_heatmap function. For continuous data like A_260/280_ OD ratio, DNA concentration, OTU richness, and Shannon Diversity Index, distribution was tested using Shapiro-Wilks test while variance was tested using Bartlett’s test. Normal distributed data was expressed by mean values and analyzed using ANOVA followed by Tukeys post-hoc test, while data that was not normal distributed or did not have equal variances, was expressed as median values and analyzed using Kruskal-Wallis Test followed by Dunn’s post hoc test. Differences were considered statistical significant for p < 0.05.

### Ethical approval

The study protocol was reviewed by the Regional Ethical Committee of Northern Denmark. Since no personal information were collected from study participants and no intervention was performed, the Ethical Committee judged that no further approval was required.

The study was carried out in accordance to the guidelines provided by the Ethical Committee of Northern Denmark concerning anonymized biological material.

## Electronic supplementary material

Supplementary images

Dataset 1

Dataset 2

## Data Availability

Sample information (Supplementary_metadata) and OTU-tables (Supplementary_Otutable) generated during sequencing and used for bioinformatics are available at the Dryad Digital Repository: doi:10.5061/dryad.61r43kd.
