# Supplementary material for: Interpersonal Variations in Gut Microbiota Profiles Supersedes the Effects of Differing Fecal Storage Conditions
Source: Sci Rep. 2018 Nov 26;8:17367. doi: 10.1038/s41598-018-35843-0 (PMC6255890; doi:10.1038/s41598-018-35843-0)
Supplement: Supplementary file 1 — Supplementary images [file 41598_2018_35843_MOESM1_ESM.pdf]

Supplementary images

Title: Interpersonal Variations in Gut Microbiota Profiles Supersedes the Effects of Differing Fecal Storage Conditions.

Authors:
Caspar Bundgaard-Nielsen[1,2], Søren Hagstrøm[1,2,3], Suzette Sørensen[1,2] *

Affiliations:
[1] Centre for Clinical Research, North Denmark Regional Hospital, Hjørring, Denmark
[2] Department of Clinical Medicine, Aalborg University, Aalborg, Denmark.
[3] Department of Pediatrics, Aalborg University Hospital, Aalborg, Denmark
* Corresponding author: Suzette.soerensen@rn.dk

1.1 Rarefication curve

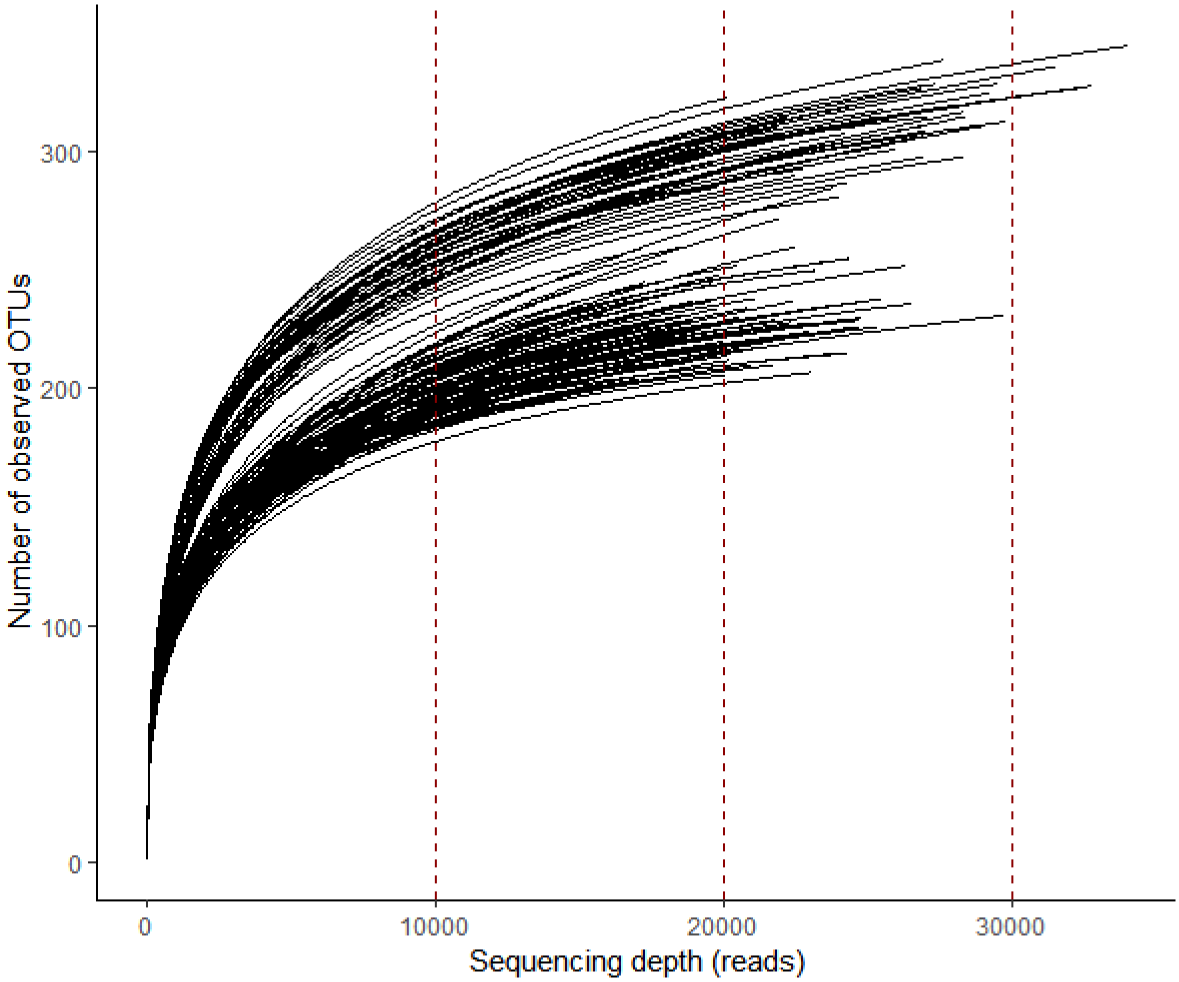

Generated in R through the Rstudio IDE, in ampvis2 package v.2.3.11. Note generation of new OTUs had started to plateu following end of sequencing. More than 10.000 reads were obtained in all samples.

1.2 A comparison of triplicates and individual samples

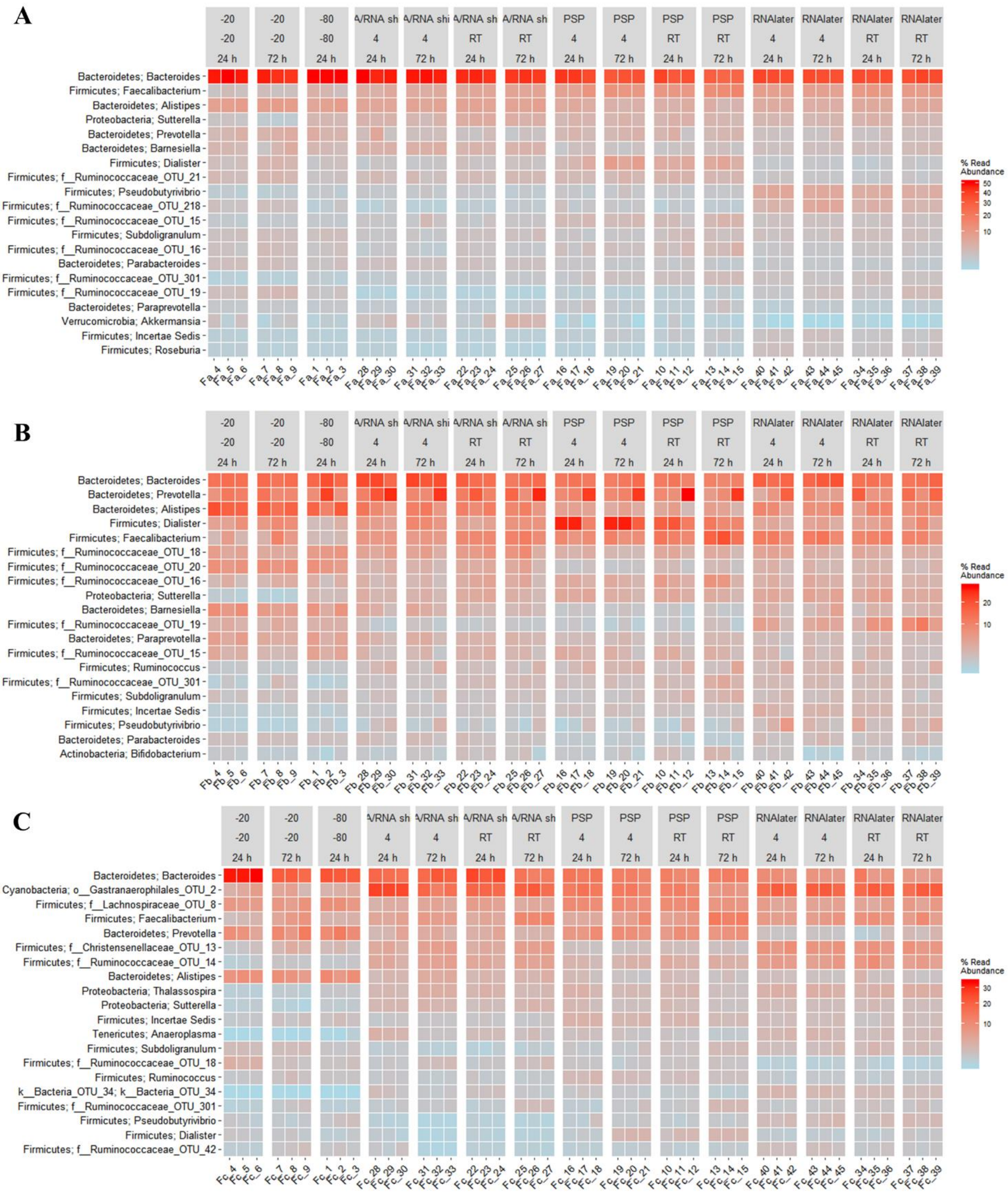

For each donor, the heatmap is facetted by each setup, with the bacterial composition of each of the three triplicates being compared side-by-side. All triplicates are highly similar.

1.3 Gel electrophoresis

DNA integrity of all fecal samples, following DNA extraction, were evaluated using agarose gel electrophoresis. The following charateristics for the electrophoresis were used:

- 1 % agarose gel in TBE buffer
- 1:10,000 concentration of Gelred Nucleic Acid Gel stain (Biotium)
- 100 V for 3 hours and 40 minutes
- GeneRule 1kb plus DNA Ladder (Thermo Scientific, see the image to the right).

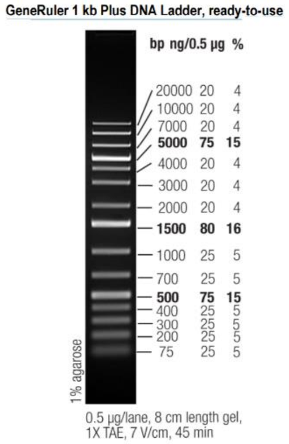

For each replicate, for each separate donor, the following setup was used for load the gel:

1	2	3	4	5	6	7	8	9	10	11	12	13	14	15	16
Ladder	-80 °C	-20 °C - 24h	-20 °C - 72h	PSP, RT - 24h	PSP, RT - 72h	PSP, 4 °C - 24h	PSP, 4 °C - 72h	DNA/RNA shield, RT - 24h	DNA/RNA shield, RT - 72h	DNA/RNA shield, 4 °C - 24h	DNA/RNA shield, 4 °C - 72h	RNA-later, RT - 24h	RNA-later, RT - 72h	RNA-later, 4 °C - 24h	RNA-later, 4 °C - 72h

Following electrophoresis, the gels were visualized using a SynGene G-boks with automatic exposure. A subsequent manual overexposure of 3 seconds was used, to improve visualizing DNA integrity.

The resulting gels can be seen on the next three pages.

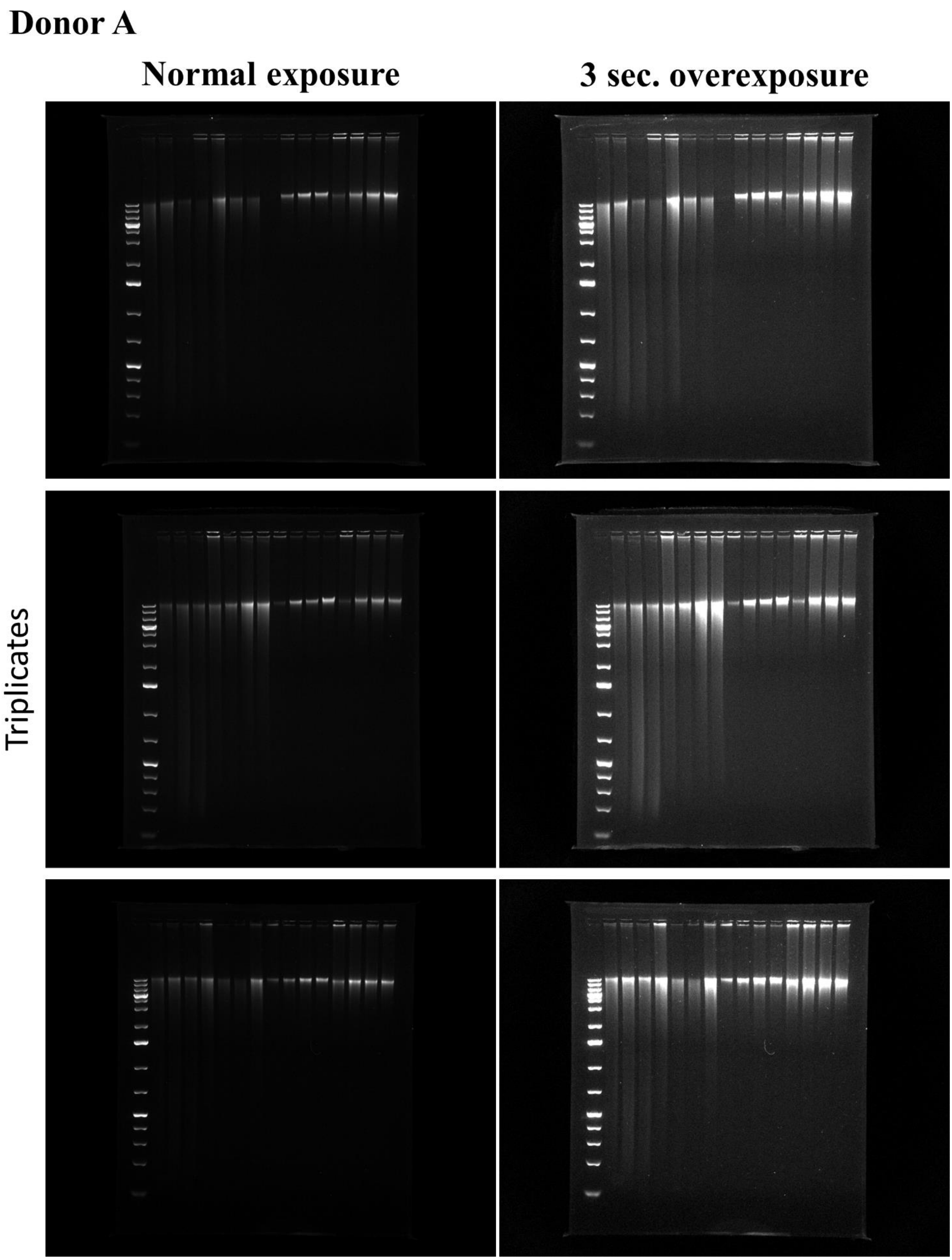
Donor A
Normal exposure
3 sec. overexposure
Triplicates

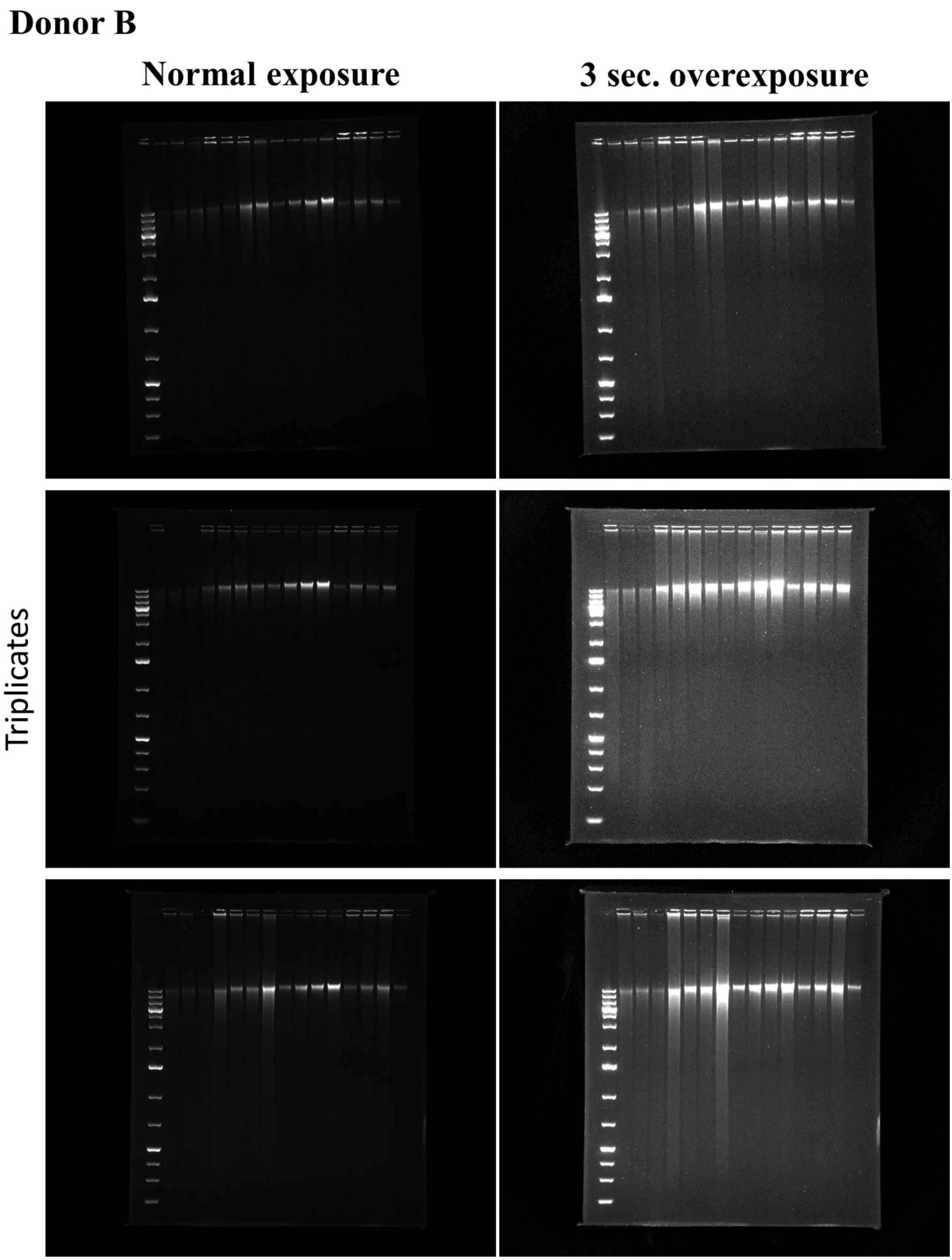
Donor B
Normal exposure
3 sec. overexposure
Triplicates

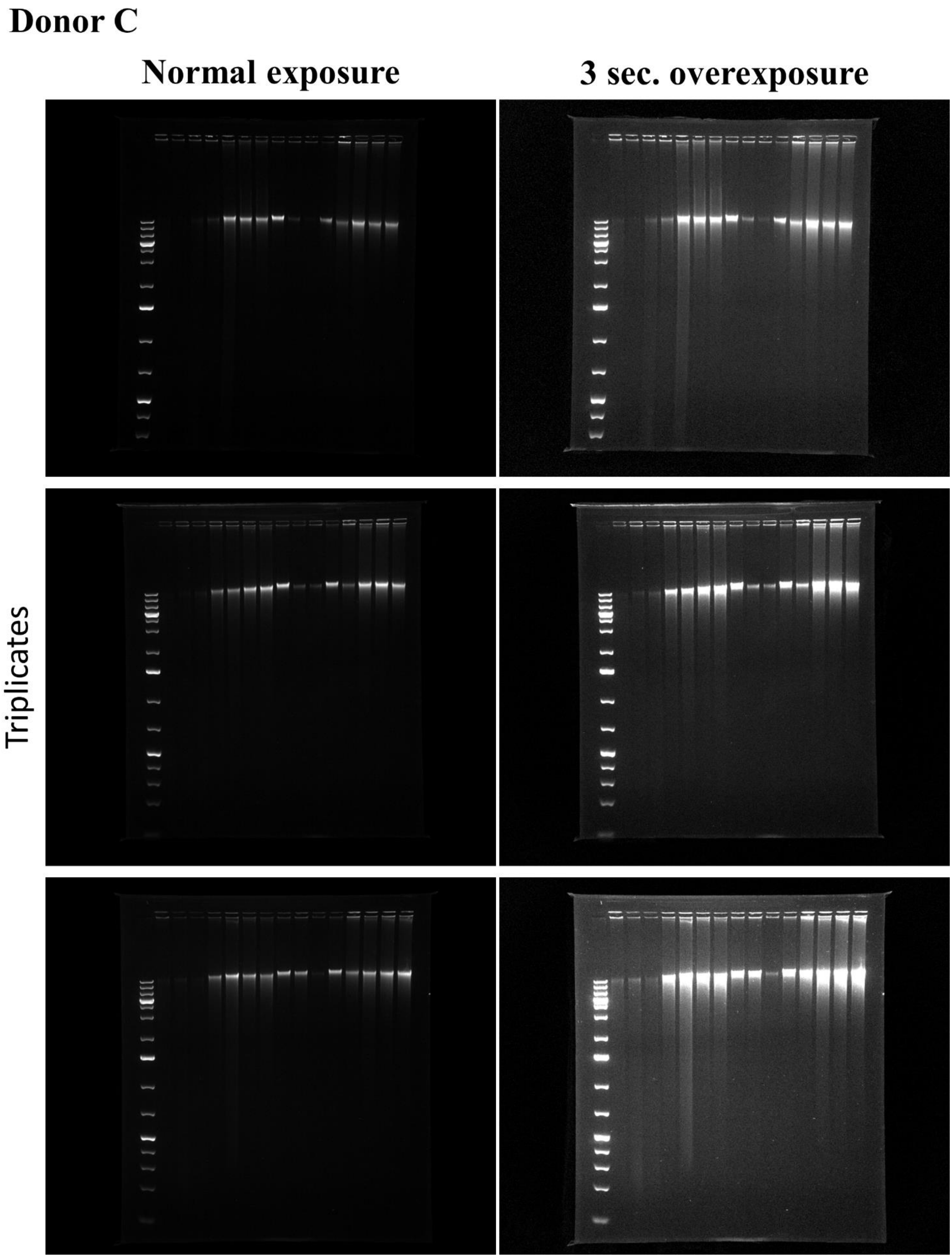
Donor C
Normal exposure
3 sec. overexposure
Triplicates
